# Inflammatory Chemokines in Atherosclerosis

**DOI:** 10.3390/cells10020226

**Published:** 2021-01-25

**Authors:** Selin Gencer, Bryce R. Evans, Emiel P.C. van der Vorst, Yvonne Döring, Christian Weber

**Affiliations:** 1 Institute for Cardiovascular Prevention, Ludwig-Maximilians-University, 80336 Munich, Germany; selin.gencer@med.uni-muenchen.de (S.G.); Van_der_Vorst@med.uni-muenchen.de (E.P.C.v.d.V.); yvonne.doering@med.uni-muenchen.de (Y.D.); 2 Department of Angiology, Swiss Cardiovascular Center, Inselspital, Bern University Hospital, University of Bern, 3010 Bern, Switzerland; bryce.evans@insel.ch (B.R.E.);; 3 German Center for Cardiovascular Research (DZHK), Partner Site Munich Heart Alliance, 80336 Munich, Germany; 4 Interdisciplinary Center for Clinical Research (IZKF), Institute for Molecular Cardiovascular Research (IMCAR), RWTH Aachen University, 52074 Aachen, Germany; 5 Department of Pathology, Cardiovascular Research Institute Maastricht (CARIM), Maastricht University, 6229 ER Maastricht, The Netherlands; 6 Department of Biochemistry, Cardiovascular Research Institute Maastricht (CARIM), Maastricht University Medical Centre, 6229 ER Maastricht, The Netherlands; 7 Munich Cluster for Systems Neurology (SyNergy), 80336 Munich, Germany

**Keywords:** chemokines, inflammation, atherosclerosis, mouse models, chemokine receptors, cardiovascular disease

## Abstract

Atherosclerosis is a long-term, chronic inflammatory disease of the vessel wall leading to the formation of occlusive or rupture-prone lesions in large arteries. Complications of atherosclerosis can become severe and lead to cardiovascular diseases (CVD) with lethal consequences. During the last three decades, chemokines and their receptors earned great attention in the research of atherosclerosis as they play a key role in development and progression of atherosclerotic lesions. They orchestrate activation, recruitment, and infiltration of immune cells and subsequent phenotypic changes, e.g., increased uptake of oxidized low-density lipoprotein (oxLDL) by macrophages, promoting the development of foam cells, a key feature developing plaques. In addition, chemokines and their receptors maintain homing of adaptive immune cells but also drive pro-atherosclerotic leukocyte responses. Recently, specific targeting, e.g., by applying cell specific knock out models have shed new light on their functions in chronic vascular inflammation. This article reviews recent findings on the role of immunomodulatory chemokines in the development of atherosclerosis and their potential for targeting.

## 1. Introduction

Chemokines are a large class of secreted cytokines with the capability of inducing cellular migration by forming chemoattractant gradients, a process also known as chemotaxis [1]. This special ability allows chemokines to perform central tasks in the development and maintenance of the immune system as well as the execution of dynamic inflammatory processes, such as immune cell recruitment. Moreover, chemokines are of prime importance in embryogenesis and a number of vital cellular functions including, but not limited to, proliferation, survival and differentiation [2]. Besides their fundamental benefits, chemokines are recognized to play key roles in the pathogenesis of inflammatory and autoimmune diseases as well as tumor progression. This review will focus on the diverse roles of chemokines in atherosclerosis, a chronic inflammatory disease of the vessel wall that lays the foundation to cardiovascular diseases (CVDs).

Atherosclerosis is a multifaceted disease, which develops and progresses over a long period of time and eventually leads to severe harm in the vascular tissue. It initially arises due to a damage to the endothelial lining of large vessels triggered by pathogenic factors, such as hyperlipidemia and hemodynamic shear stress [3]. Damaged endothelium becomes activated and initiates an inflammatory response in order to recruit immune cells to the site of injury. In order to do so, endothelial cells (ECs) secrete chemokines, such as CXCL1, to be sensed and tracked back to the site of injury by the immune cells. The aim of these immune cells, primarily classical monocytes, is to enter the sub-endothelial space to subsequently eliminate the factors that are damaging the endothelium, such as oxidized low-density lipoprotein (oxLDL). It is well known that LDL breaches the arterial wall in cases of excessive cholesterol presence in the blood and it becomes oxidized in the sub-endothelial space. This triggers an inflammatory response in the vascular endothelium leading to a cascade of atherosclerotic events [4]. Invasion of the arterial wall by leukocytes is one of the key drivers of atherosclerotic lesion development and it involves several stages, such as recruitment, adhesion and trans-endothelial infiltration of the cells. These processes are aided by a variety of chemokines, like CXCL1 and CCL2. Post-infiltration, monocytes differentiate into macrophages in the intima in order to engulf and clear oxLDL. The latter has also been shown to be regulated by other chemokines, such as the CXCL12 [5]. Lipid engulfing macrophages eventually become overloaded and turn into foam cells, which form the fatty streaks observed in atherosclerotic lesions. Hence, chemokines widely contribute to many aspects of the pathogenesis and progression of atherosclerotic lesions [6]. Besides the initiation and growth of the lesions, chemokines can also modulate the stability of the lesions. Smooth muscle cells (SMCs) produce collagen and elastin, which form a so-called ‘fibrous cap’ around the lesions in order to keep the lesions stable and to prevent rupture. In addition to lesional collagen content, fibrous cap thickness is a good indicator of lesion stability and it can be influenced by chemokines, such as CXCL10, among other factors, like matrix metalloproteinases [7]. Thinning of the fibrous cap and rupturing of the lesions may lead to thrombus formation and thereby occlude arteries resulting in the disruption of blood flow to downstream tissues and thereby lead to ischemia related severe clinical consequences, such as heart attack and stroke. Understanding in what ways chemokines contribute to the progression of atherosclerosis may help researchers identify and establish key steps to effectively prevent or treat CVD. Nonetheless, due to the abundance of the chemokines and chemokine receptors in cells, as well as the richness of their functions, this task implicates enormous complexity. It is important to note that a chemokine/receptor axis may be beneficial in various aspects whilst contributing to atherosclerotic events. In this regard, treatment approaches targeting blockade of certain chemokine axis may introduce unwanted side-effects and cell specific targeting should be one of the main future roads to explore.

**Figure 1:**
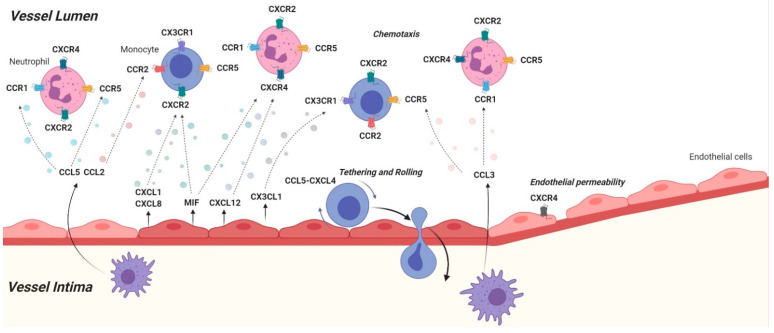
Schematic and simplified overview of the effects that chemokine-chemokine receptor axis have on leukocyte recruitment. Various chemokines and chemokine receptors are visualized that play a role in leukocyte recruitment. Activated endothelial cells secrete a wide variety of chemokines that attract leukocytes like monocytes and neutrophils towards the vessel wall in a process called chemotaxis, followed by tethering, rolling and transmigration. Furthermore, activated macrophages secrete chemokines that stimulate this leukocyte recruitment process and it has been shown that chemokine receptors influence endothelial permeability, which is an important aspect for leukocyte transmigration (Created with Biorender.com).

**Figure 2:**
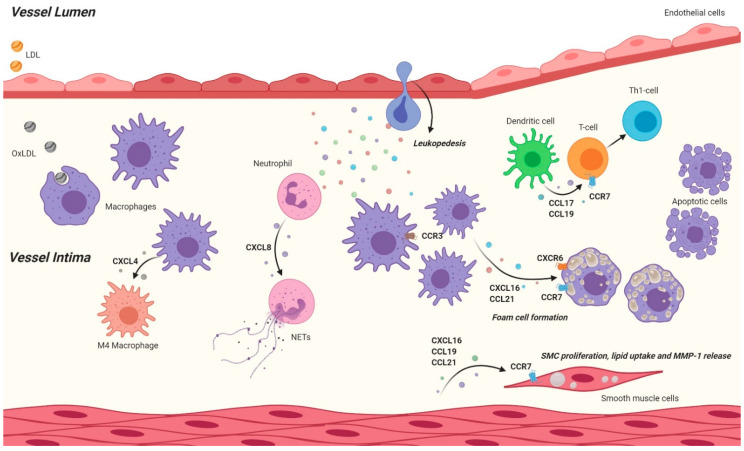
Schematic and simplified overview of the effects that chemokine-chemokine receptor axis have on atherosclerotic processes inside the vessel wall. In the vessel wall, various chemokines and chemokine receptors play an important role in cellular processes. Secreted chemokines contribute to the leukopedesis and stimulates foam cell formation. Furthermore, chemokines contribute to macrophage polarization and the formation of neutrophil extracellular traps (NETs). Besides effects on myeloid cells, also lymphocytes are affected as dendritic cell mediated T cell activation is stimulated by chemokines. Finally, chemokine and chemokine receptors contribute to smooth muscle cell (SMC) proliferation, lipid uptake and release of matrix metalloproteases. (Created with Biorender.com).

Chemokines can be classified into four structural groups based on their cysteine residue order: CC, CXC, CX_3_C, and XC [8]. They induce cell responses by signaling through seven-transmembrane G-protein coupled receptors (GPCRs), which are known as the ‘classical’ chemokine receptors, as well as atypical chemokine receptors (ACKRs), which are similar to GPCRs but cannot signal through G-proteins [9]. The role of chemokines and their receptors in atherosclerosis is being investigated via several methods including genetic and pharmacologic manipulations of these molecules. For example, vascular injury induced or diet-induced atherosclerotic mouse models, such as apolipoprotein-E deficient (ApoE^−/−^) or low-density lipoprotein receptor deficient (Ldlr^−/−^) mice on Western type diets are applied. These mice are also genetically modified resulting in lack of certain chemokines or chemokine receptors (systemically or in a cell-specific manner) in order to investigate the role of the target molecules in the context of the atherosclerosis. Another approach serving the same purpose is pharmacological inhibition or enhancement of the activity of the chemokines and their receptors. Several studies applying these methods have shown significant roles of certain chemokines in atherosclerosis; here we will provide a concise overview (Figure 1 and Figure 2) of these findings and list important chemokine/receptor axes in the context of atherosclerosis (Table 1). 

### 1.1. CCL2-CCR2

CCL2, also known as the monocyte chemoattractant protein-1 (MCP-1), is a potent inflammatory chemokine and signals through its receptor CCR2 [10]. Its acute inflammatory effects were studied in an in-vivo model using CCL2-deficient mice with a thioglycollate induced peritonitis [11]. Whilst the control mice exhibited significant increases (as high as a six-fold surge) in the numbers of monocytes and macrophages in their peritoneal cavities, CCL2-deficient mice displayed a comparatively reduced cell recruitment (two-fold) in response to the inflammation. It was also observed that the surge in the peritoneal cavity cell numbers of the control mice was mainly due to monocytes and macrophages with a small number of neutrophils and eosinophils, whereas CCL2-deficient mice seemed to lack monocyte and macrophage recruitment whereas showing a similar increase in neutrophils and eosinophils as observed in control mice. 

Strong evidence as early as 1998 established a pro-atherosclerotic role of the CCL2/CCR2 axis. An in-vivo atherosclerosis study using CCL2-deficient Ldlr^−/−^ mice on a long term high cholesterol diet (≥12 weeks) revealed significantly reduced lipid deposition in the aortas of CCL2-deficient mice along with fewer macrophages infiltrating into the aortic walls compared to the control mice [12]. Confirming these observations, another study showed that CCR2-deficient ApoE^−/−^ mice exhibited significantly reduced lesion formation as well [13]. The authors likewise showed that the aortas of CCR2-deficient mice contained less macrophages compared to control mice and concluded that the CCL2–CCR2 axis was important in the monocyte/macrophage accumulation in the vessel wall. This conclusion was backed up in 1999 via similar findings from Dawson et al [14]. and another study in 2003 revealed direct evidence that leukocyte specific CCR2-deficiency massively reduced atherosclerotic lesions via a CCR2-deficient bone marrow cell transplantation study in ApoE3-Leiden mice [14,15]. Such reproducible results through different types of atherosclerotic mouse models well established that the CCL2-CCR2 axis is a strong driver of atherosclerosis and makes it an important target to study in atherosclerosis.

Recently, researchers discovered that myeloid cell recruitment to atherosclerotic lesions was regulated in a circadian rhythmic fashion and that the myeloid cells deposited CCL2 onto the arterial wall in order to facilitate adhesion to the endothelium [16]. Ref. [16] According to the 4-week high-fat diet study, pharmacological blockade of CCR2 via its antagonist RS102895 in a timed manner, targeting the peak of CCL2-CCR2 driven myeloid cell adhesion to the arterial wall, abolished the adhesion and resulted in decreased atherosclerotic lesion sizes along with diminished lesional macrophage accumulation. Besides these roles of CCL2, others have also shown its importance in neointimal hyperplasia formation [17]. In a cuff-induced arterial injury study, CCR2-deficient mice exhibited significantly less neointimal hyperplasia compared to control mice [18]. A similar study further confirmed that arteries from CCR2 deficient mice showed above 60% reduction in the intimal area as well as intima/media ratio compared to control mice after four weeks of injury [19]. Hence, CCL2 plays an essential role in monocyte trafficking and results suggest that the CCL2-CCR2 axis may be an important target to treat acute arterial injury and complications, such as restenosis in CVD patients. 

### 1.2. CCL3

Neutrophils are potent inflammatory short acting cells associated with increased intimal apoptosis and a proinflammatory phenotype [20]. It is thought that neutrophil infiltration induces plaque destabilization due to enhanced inflammation, intimal apoptosis, necrotic core formation, and matrix degradation. CCL3 interacts with chemokine receptors CCR4, CCR1 and CCR5, of which the latter two have been implicated in atherogenesis and are found on neutrophils [21]. Activated macrophages are the main source of CCL3, however it can also be released by activated platelets, neutrophils, and mast cells [22,23]. A previous study has shown that CCL2 and CCL3 induce an inflammatory cascade regulating firm adherence and infiltration of neutrophils [24]. Thus, CCL3 may play an important role in neutrophil recruitment and the development of atherosclerosis. In a bone marrow transplant experiment where irradiated Ldlr^−/−^ mice were transplanted with either *CCL3*^−/−^ or Ldlr^−/−^ bone marrow and fed a western diet (WD) for 12 weeks, resulted in reduced CCL3 release in response to LPS treatment suggesting leukocytes are a critical source of CCL3. Furthermore, hematopoietic CCL3-deficiency significantly reduced aortic lesion formation by 31%, moreover neutrophil adhesion to and presence in plaques was significantly attenuated [25]. This demonstrates that under inflammatory conditions leukocyte-derived CCL3 can induce neutrophil chemotaxis toward the atherosclerotic plaque, thereby accelerating lesion formation.

However, the role of CCL3 in atherosclerosis may be dependent on the various receptors as deficiency of CCR1 in ApoE^−/−^ mice fed a WD for 10 to 12 weeks led to accelerated atherosclerosis and greater plaque size and infiltration of T lymphocytes [21]. However, functional deficiency of CCR5 in ApoE^−/−^ mice was shown to reduce atherosclerotic lesion development, and plaques contained less macrophages and T-cells [21]. An in vivo study has investigated the therapeutic potential of targeting CCL3 using atorvastatin in ApoE^−/−^ mice [26]. Three groups were investigated, chow diet feeding only and two WD fed groups (16 weeks) of which one was treated with atorvastatin. The study found that atorvastatin inhibited the 5-LO pathway and downregulated the gene and protein expression of CCL3 significantly attenuating atherosclerotic lesions, in ApoE^−/−^ mice. Taken together, deficiency of CCL3 reduces the atherosclerotic burden, suggesting that blocking of CCL3 maybe using a monoclonal antibody may be a promising therapeutic treatment.

### 1.3. CCL5-CCR1/CCR5/CCR3

The chemokine CCL5 is also referred to as RANTES and it is known to bind multiple receptors: CCR1, CCR3 as well as CCR5 [27]. In 2001, platelet derived deposition of CCL5 on activated endothelium was shown to elicit monocyte arrest suggesting a pivotal role for CCL5 in the development of atherosclerotic lesions [28]. This finding was further endorsed through a study by Schober et al. disclosing that the monocyte arrest driven by platelet-derived CCL5 required platelet P-selectin [29]. Moreover, the authors showed that the systemic inhibition of CCL5 hindered neointima formation as well as macrophage infiltration in a wire-induced injury model *in ApoE^−/−^* mice. Soon after, a study investigated the effects of systemic CCL5 antagonism via Met-RANTES for 14 weeks in a diet-induced mouse atherosclerosis [30]. These findings demonstrated a reduction in atherosclerotic lesions observed in the aortic roots as well as the thoracoabdominal aortas along with a decline in leukocyte infiltration in Met-RANTES treated mice. The lesions of the control mice were more abundant in macrophage foam cells as well as T lymphocytes. Altogether, these findings established that CCL5 fuels atherogenesis by supporting monocyte arrest on the endothelium and immune cell infiltration into the lesions. 

CCL5 is also known to heteromize with the chemokine CXCL4, which is also platelet derived and deposited onto vascular endothelium [31]. In human atherosclerotic lesions, CXCL4 was found to correlate with lesion severity and platelet specific deletion of *Cxcl4* was found to decrease atherosclerotic lesions in mice [32,33]. A detailed study by Huo et al. nicely disclosed that platelet derived CCL5 and CXCL4 were delivered to the surface of vascular endothelium as well as monocytes [34]. Activated platelets supported leukocyte adhesion to the arterial wall via stimulation of vascular cell adhesion molecule-1 (VCAM-1) and thereby promoted atherosclerosis. Consistent with these findings, another study showed that the heterophilic interaction of CCL5 and CXCL4 promoted monocyte arrest by treating monocytes with the supernatants of activated platelets [35]. Interference with the CCL5-CXCL4 heterodimer through a cyclic peptide, named MKEY, was proven to be beneficial in an mouse model of myocardial infarction as the blockade decreased leukocyte recruitment as well as the release of neutrophil extracellular traps (NETs) [36]. The study further reported decreased inflammation and infarction sizes which was associated with reduced monocyte and neutrophil infiltration into the infarction areas. Additionally, CCL5 can form heteromers with neutrophil-borne human neutrophil peptide 1 (HNP1), which was also shown to drive monocyte adhesion through CCR5 [37].

Whilst the pro-atherosclerotic role of CCL5 and its heterodimers is clear, studies dissecting the roles of the CCL5 receptors CCR1 and CCR5 in vascular inflammation show contradicting results. A diet induced atherosclerotic mouse model study on an ApoE^−/−^ background showed that genetic deletion of CCR5 resulted in reduced atherosclerotic lesions along with a more stable plaque phenotype [21]. Within the same study, however, CCR1 deficiency resulted in increased lesion areas in mice. Nevertheless, others report significantly decreased plaque sizes in CCR1-deficient ApoE^−/−^ animals after 4 weeks WD [38]. Hematopoietic CCR1 deficiency on Ldlr^−/−^ background revealed a 70% increase in atherosclerotic lesion sizes in the thoracic aorta of mice after long term high fat diet feeding [39]. Ref. [39] These findings indicate that the receptor CCR5 drives atherosclerosis, whereas the receptor CCR1 is debatable. The atherosclerotic effects of CCR5 were further demonstrated in a recent, randomized pilot study with human patients [40]. An antagonist of CCR5, maraviroc, was administered in human immunodeficiency virus (HIV) patients for 24 weeks and several markers of atherosclerosis were measured as follows: brachial flow-mediated dilation (bFD), carotid-femoral pulse wave velocity (cFPWV) and carotid intima-media thickness (cIMT). Further factors, such as systemic inflammatory markers and monocyte & platelet activation were also examined in the study. Results revealed that CCR5 antagonism through maraviroc significantly improved several markers of atherosclerosis, such as bFD, cFPWV, and cIMT, whereas systemic inflammation and monocyte activation were not altered significantly through the treatment. Furthermore, the antagonism study showed a significant improvement in the vascular competence, which is described as the ‘ratio of circulating endothelial microparticles (EMPs) to endothelial progenitor cells (EPCs)’. The authors of this study concluded that CCR5 antagonist maraviroc is protective against important cardiovascular risk markers, such as endothelial dysfunction and arterial stiffness. Despite several limitations within the study, these results indicate that CCR5 inhibition may be a promising target in CVD research and further studies are needed in order to establish a better understanding of its effects. Apart from CCR5 and CCR1, CCR3 has been shown to be overexpressed in CD68+ macrophage rich areas of human atherosclerotic lesions, however there is not sufficient information regarding its roles in atherosclerosis, especially through interaction with CCL5 [41].

### 1.4. CCL17

CCL17 is elevated in patients with Atopic dermatitis (AD) of all ages and patients with AD present a greater risk of developing CVD [42,43]. CCL17 has been observed in advanced human and mouse atherosclerotic lesions [44]. The mostly DC-derived chemokine CCL17 activates the chemokine receptor CCR4 and was first thought to preferentially promote T cell responses with a Th2 bias. However, it is now understood that CCL17, via CCR4, can also attract effector/ memory T cells of the Th1 subtype and also regulatory T cells (Tregs) [45]. In atherosclerosis-prone ApoE^−/−^ mice fed a high fat diet for 4 weeks, CCL17 deficiency attenuated atherosclerosis in a Treg-dependent manner. Hence, vascular CCL17 may recruit T cells along with other proinflammatory molecules expressed in plaques, as less CD3^+^ T cells were found in lesions of mice lacking CCL17 [44]. Moreover, adoptive transfer of labeled CD4+ T cells into atherosclerotic ApoE^−/−^ or CCL17-deficient ApoE^−/−^ mice resulted in more CD4^+^ T cells homing to aortas of Apoe^−/−^ mice [44]. Similarly, Foxp3^+^ Treg expansion but reduced secretion of IL-12 and IL-23 was also observed in an inflammatory colitis model if these animals lacked CCL17 [46]. Hence, CCL17^+^ DCs in atherosclerotic promote recruitment of inflammatory T cells to the vessel wall while restraining Treg maintenance at sites of inflammation and in lymphatic organs. In support of this, antibody specific blocking of CCL17 resulted in Treg expansion and reduced atheroprogression [44]. These data suggests that CCL17 DCs may regulate homeostatic mechanisms in T cells primarily in lymphoid tissue and regulate Treg homeostasis in atherosclerosis. A structure-function analysis demonstrated that CC-type heterodimers can improve chemokine activity, whereas CXC-type heterodimers can inhibit these functions. Hence, CCL5-CCL17 heterodimers were shown to drive lung injury and atherosclerosis, however this effect was reversed when a CCL5-derived peptide inhibitor was used [47]. This CCL5-derived peptide formed a heterodimer with CXCL12, thereby disrupting the CCL5-CCL17 heterodimer and mimicked the results of a *CCL17^eGFP/eGFP^* mouse model. This demonstrates the atheroprogressive role of CCL17 and suggests that disruption of chemokine heterodimerization or blocking CCL17 could be a potential therapeutic target [47].

In line a study of 158 non-coronary artery disease (CAD) patients and 813 CAD patients showed that CAD patients had higher serum CCL17 levels compared to patients without CAD [48]. Another study with 794 patients with CAD and 153 without CAD demonstrated that the T allele at rs223828, which is located in the intron of the *CCL17* gene, corresponded with increased CCL17 serum levels and increased CAD risk [49]. Furthermore, a luciferase assay showed that the rs223828T allele enhances the CCL17 promoter activity and a ChIP assays demonstrated that the activator protein-1 (AP-1) was preferentially recruited to the rs223828 T allele compared with the C allele. The latter fits the notion that AP-1 deficiency protects against the development of atherosclerosis in hypercholesterolemic mice Thus, it is suggested that the CCL17 single-nucleotide polymorphisms rs223828 correlates with increased risk of CAD, via AP-1 activation.

### 1.5. CCL19, CCL21/CCR7

A clinical study demonstrated that CCL19 and CCL21 was up regulated in carotid atherosclerosis in 158 patients compared to a control of 20 patients, suggesting an important role for these chemokines in atherosclerosis [50]. CCL19 mRNA has also been observed in human atherosclerotic lesions proposing it has a role in atherosclerosis [51]. CCL19 is secreted by DCs, while both CCL21 and CCL19 are secreted from the endothelium [52,53]. Both of these chemokines share the same receptor CCR7, which is expressed by T cells, naive B cells, DCs and NK cells [54,55]. Regulation of DC maturation is essential to balance the protective T cell response and immunopathology. CCL19 induces a maturation of DCs, resulting in the upregulation of costimulatory molecules and the production of pro-inflammatory cytokines which in turn enhances T cell proliferation [56]. Furthermore, CCL19-induced DCs preferentially induce a Th1 rather than a Th2 response. The upregulation of a Th1 response further drives the progression of atherosclerosis [56,57]. 

Isolation of DCs from mice deficient in CCL19 and CCL21 (plt/plt) had only a partially mature phenotype, stressing the importance of these chemokines for full DC maturation in vivo [56]. However, in vitro experiments showed that oxLDL reduced both gene and protein expression of CCR7 on DCs and CCL21 protein expression on human microvascular endothelial cells (HMECs) [58]. This suggests that retention of DCs in atherosclerotic plaques correlates with the downregulation of chemokines and their ligands, known to regulate DC migration. These DCs may then drive the inflammatory response in atherosclerosis and enhance the risk for plaque rupture. Thus, modulation of DC maturation may prove to be a promising therapeutic treatment.

Examinations in vitro demonstrated the ability of CCL21 to promote lipid accumulation in macrophages while CCL19 induces SMC proliferation and matrix metalloprotein-1 expression [50]. This suggests that these chemokines may also directly contribute to the development of atherosclerosis. CCL19 stimulated HUVECs for example facilitate monocyte adhesion and migration, whereas CCL21 only induced migration but not adhesion [59]. The same study also showed that clinically achievable concentrations of atorvastatin suppressed CCL19/CCL21/CCR7 expression and inhibited CCL19/ CCL21-induced monocyte adhesion and migration [59]. However further research is required in vivo to determine the CCL19/CCL21/CCR7 role in the recruitment and infiltration of monocytes in atherosclerosis and to determine its therapeutic potential.

The role of CCL19 and CCL21 in experimental atherosclerosis in mice is still disputed as results from different studies are contradictory. In an ApoE^−/−^CCR7^−/−^ mouse model, were mice were fed a WD for 8 weeks, the lesion size was increased due to an increase in T cell infiltration into the atherosclerotic lesions and the blood, bone marrow, and spleen [60]. In an adoptive transfer experiment T cells from ApoE^−/−^CCR7^−/−^ mice migrated poorly into lymph nodes but better into mouse aortas compared to T cells from CCR7-competent animals, suggesting that CCL19 and CCL21 facilitate T cell homing to lymphatic orangs thereby exhibiting atheroprotective functions [60]. However, in an Ldlr^−/−^ mouse model, where mice were fed a high-cholesterol diet for 12 weeks, the adoptive transfer of wild-type T cells resulted in atheroprogression in Ldlr^−/−^CCR7^−/−^ mice while transfer of CCR7-deficient T cells into Ldlr^−/−^CCR7^−/−^ mice did not result in increased lesion formation [61]. This suggests that CCR7 is atheroprogressive by mediating CCR7-dependent T-cell priming in secondary lymphoid organs and CCR7-dependent T cell homing to the atherosclerotic lesion. In a bone marrow transplant experiment of CCL21-deficient bone marrow into Ldlr^−/−^ animals it was further demonstrated that CCL21 coordinates the homing of leukocytes into atherosclerotic lesions, whereas CCL19 influences the activation of leukocytes, lipid uptake of macrophages and foam-cell formation [62]. Taken together studies in ApoE^−/−^ animals reveal a protective role of CCR7 while reports in Ldlr^−/−^ mice report an atheroprogressive role of this receptor. These conflicting results may be due to the different mouse models used or due to systemic knockout of the CCR7 in ApoE^−/−^ versus adoptive transfer and bone marrow transplantation in Ldlr^−/−^ animals. Further investigations on the role of these chemokines in vivo are needed.

### 1.6. CXCL1-CXCR2

Chemokines are involved in all stages of atherosclerosis with various roles. As mentioned above, endothelial dysfunction is a major starting point of lesion formation. Once the vascular endothelium is activated, it secretes inflammatory markers to recruit immune cells. The chemokine axis CXCL1(GRO-alpha or KC)/CXCR2 was shown to be especially important in the initiation phase of atherosclerotic lesion development. In 1994, Schwartz et al. suggested that a GRO homologue was involved in monocyte binding to minimally modified LDL (MM-LDL) stimulated human aortic endothelial cells (HCAECs) [63]. The authors reported that the mRNA expression of CXCL1 levels as well as the surface expression of a protein binding to CXCL1-antibodies were significantly increased in HCAECs upon MM-LDL exposure. Moreover, administration of an antibody against CXCL1 inhibited the monocyte binding to HCAECs and interestingly heparin treatment on ECs (releasing heparin-bound molecules from the cell surface) resulted in the inhibition of both CXCL1 expression as well as monocyte binding. Collectively, these results suggested that a specific type of CXCL1 was expressed on the surface of ECs and mediated monocyte binding to the activated endothelium. This role of GRO/CXCL1 was later confirmed in a study using a flow chamber system, which facilitates ex-vivo perfusion studies on the carotid arteries extracted from ApoE^−/−^ mice that are susceptible to developing atherosclerotic lesions. The authors reported that CXCL1 significantly governed monocyte arrest on endothelium [64]. Both anti-CXCL1 antibody blocking, as well as the blocking of its receptor CXCR2, abolished the observed monocyte arrest on the endothelium suggesting that this axis plays an important role in monocyte accumulation on atherosclerotic endothelium.

These findings were carried forward with an in-vivo model study, which investigated the impact of CXCL1 on atherosclerosis using CXCL1-deficient Ldlr^−/−^ mice. After 16 weeks of high fat diet CXCL1-deficient Ldlr^−/−^ mice exhibited significantly less atherosclerotic lesions in their aortas as well as aortic valves compared to control mice [65]. Moreover, immunohistochemical staining of the aortic valves revealed diminished macrophage accumulation in the lesions of CXCL1 deficient mice. The study additionally examined the role of leukocyte specific CXCL1 via bone marrow transplantation. Hematopoietic deficiency of CXCL1 did not alter atherosclerosis, suggesting that the atherosclerotic effects of CXCL1 were attributable to non-hematopoietic cells. Interestingly, they had previously shown that the deficiency of the CXCL1 receptor CXCR2 in the bone marrow of Ldlr^−/−^ mice resulted in significantly reduced atherosclerotic lesions along with decreased lesional macrophage accumulation at the end of a 16-week high fat diet, suggesting a pro-atherosclerotic role for leukocyte specific CXCR2 [66]. 

Another study assessing ApoE^−/−^ mice on eight weeks of high fat versus chow diet reported that CXCL1 levels were significantly increased in the sera of high fat diet fed mice [38]. This was associated with higher numbers of classical monocytes in the circulation of these animals together with a decrease in the bone marrow, suggesting that CXCL1 plays a key role in hypercholesterolemia induced monocytosis. The authors then studied the effects of CXCL1 neutralization with antibody injections during four weeks of high fat diet in ApoE^−/−^ mice and revealed a significant decrease in monocytosis in comparison to control mice. Furthermore, CXCL1 neutralization led to decreased atherosclerotic lesions in the aortic roots, as well as reduced monocyte and macrophage counts in the aortas of the mice [38]. These findings clearly indicate the importance of the CXCL1/CXCR2 axis in classical monocyte recruitment under hyperlipidemic conditions, which is a potent atheroprogressive event and therefore a promising therapeutic target. 

### 1.7. CXCL4

CXCL4 (also platelet factor 4, PF4) was initially identified as a product of activated platelets but is now known to be secreted by a variety of immune cells [67]. CXCL4 has a broad range of biological functions including induction of respiratory burst in human monocytes accompanied by secretion of several chemokines such as CCL3, CCL4, and CXCL8 [68]. However, in atherosclerosis CXCL4 prevents monocyte apoptosis and promotes macrophage differentiation from human peripheral blood monocytes [69]. Differentiation plays a key role in the progression of atherosclerosis or the recovery, as demonstrated by both in vitro and in vivo data. Typically macrophages exhibit two phenotypes, the M1 phenotype promotes atherosclerosis whereas the M2 plays a more protective role [70,71]. CXCL4 acts on macrophages in a unique way developing a non-characteristically macrophage phenotype, this CXCL4 lineage is referred to as M4 [72]. The CXCL4 induced macrophage phenotype shares similarities with both M1 and M2 macrophages. Specifically 375 genes were differentially expressed between M-CSF and CXCL4 induced macrophages of these, 206 genes were overexpressed in CXCL4 macrophages and are implicated in the inflammatory/immune response, antigen processing and presentation, and lipid metabolism [72]. Interestingly, M4 loses the capability to phagocytose zymosan beads. In the M4 phenotype scavenger receptors expression was reduced whereas cholesterol efflux transporters showed higher expression compared to M-CSF induced macrophages, resulting in lower LDL content. However, the role of CXCL4 on the development of foam cells in vivo has yet to be determined. 

In addition, CXCL4-induced macrophages further lack the expression of the hemoglobin scavenger receptor CD163 [73]. CD163 is a scavenger receptor for hemoglobin and hemoglobin– haptoglobin (Hb-Hp) complexes, which upregulates the enzyme heme oxygenase-1 that reportedly protects from atherosclerosis [74]. Although M4 macrophages share characteristics of both M1 and M2 they still possess an overall pro-atherogenic effect, as the deletion of the *PF4* gene encoding for CXCL4 results in reduced lesion size in atherosclerotic ApoE^−^/^−^ mice [33]. This demonstrates that CXCL4 has a significant role in the development of atherosclerosis. More research is needed to understand the role of the M4 phenotype in atherosclerosis and its potential as a therapeutic target. Furthermore, the receptor for CXCL4 is still disputed as many potential receptors have been investigated but none was conclusively identified as of today [73,75]. 

### 1.8. CXCL8

A key aspect of atherosclerosis is the oxidation of LDL which is recognized by the immune system as a danger signal and results in the release of CXCL8 [76]. CXCL8 (also IL-8) is an important inflammatory factor involved in many inflammatory responses and diseases including the development of atherosclerosis [77]. CXCR1 and CXCR2 recognize CXCL8, but CXCR2 displays higher affinity towards CXCL8 [78]. Recently, CXCL8 has been identified as a critical regulator in the function of ECs and vascular SMCs. Therefore, it is likely to participate in the development of atherosclerosis [79]. One study showed that a siRNA for the Transmembrane protein 98 (TMEM98) inhibits CXCL8 mediated leukocyte adhesion to the endothelium by down-regulating Intercellular Adhesion Molecule 1 (ICAM-1) expression on ECs. Furthermore, it also inhibits the proliferation and migration of vascular SMCs through suppressing the AKT/GSK3β/Cyclin D1 signaling pathway. These actions synergistically could reduce the development of atherosclerosis, but would require further investigation in vivo. In an in vitro study, CXCL8 enhances the expression of miR-183, which then inhibits ABCA1 expression and cholesterol efflux, suggesting that the CXCL8-miR-183-ABCA1 axis may play an transitional role in the development of foam cells in atherosclerosis [80]. Thus, CXCL8 may influence the development of atherosclerosis via the inhibition of cholesterol efflux protein ABCA1 thereby promoting the development of foam cells. Further research is needed to understand this in an in vivo setting.

An in vivo experiment assessed the potential for CXCL8 as a therapeutic target. *ApoE^−/−^* mice on high fat diet for 12 weeks were injected subcutaneously with CXCL8(3-72)K11R (G31P), a human CXCL8 analog in which the 11th amino acid (AA) lysine was substituted to arginine and the 31st AA glycine was substituted to prolineG31P [81,82]. Although mice do not possess the *CXCL8* gene they do express KC/GRO-a belonging to the GRO chemokines, and the human G31P can function in mice [83]. G31P reduced the serum concentration of LDL-C, and decreased the secretion of keratinocyte chemoattractant and the gene expression of MMP-2, MMP-9, Proliferating cell nuclear antigen (PCNA), and Myocyte Enhancer Factor 2a (Mef2a). Furthermore, G31P also inhibited the gene expressions of p-ERK, ROCK1, ROCK2, and decreased the calcium concentrations in the A7r5 cell line, in vitro. These factors inhibit the proliferation and migration of VSMCs through regulating the Rho-kinase, ERK, and calcium-dependent pathways, and therefore inhibit the development of hyperlipidemic conditions. This suggests that G31P suppresses the development of atherosclerosis by antagonizing the CXCL8 receptor. In abdominal aortic aneurysm (AAA) the CXCR1/2 antagonist DF2156A disrupted CXCL8 signaling reversing the formation of AAA, and prevented matrix degradation in the murine elastase model [84]. Patients with atherosclerosis show a higher serum level of CXCL8 and neutrophil extracellular traps (NETs) [85]. CXCL8 interacted with its receptor CXCR2 on neutrophils, leading to the formation of NETs via Src and extracellular signal-regulated kinase (ERK) and p38 mitogen-activated protein kinases (MAPK) signaling to aggravate atherosclerosis progression in vivo [85]. In summary, CXCL8 plays an important role not only in atherosclerosis but also in other vascular diseases and may be a promising therapeutic target.

### 1.9. CXCL9-CXCL10-CXCL11/CXCR3 

Increased levels of CXCL9, CXCL10, and CXCL11 can be detected in human atherosclerotic lesions throughout all stages of plaque development [6]. Endothelial cells and macrophages can release CXCL9, CXCL10 and CXCL11 all of which share the receptor CXCR3 [86]. CXCR3 activation facilitates the recruitment and homing of active Th-1 cells to the site of plaque development or rupture [86,87]. However, the different chemokines of CXCR3 have slightly different roles in T cell trafficking, as CXCL9/CXCL10 activate a different signaling cascade than CXCL11 [88]. 

CXCL10 is expressed by T cells and monocytes and facilitates T cell retention within the lesion [89]. CXCL11 is able to induce the chemotaxis of mature CXCR3+ T but not resting or naïve T cells suggesting that CXCL11 only plays a role during pro-inflammatory conditions [90]. Furthermore, CXCL11 has been found to induce the down-regulation of CXCR3 expression on T cells after they have been in contact with interferon-activated endothelial cells [91]. This could serve as an arrest signal for the activated T cells thereby limiting the inflammatory responses [92]. CXCL11 does also interact with the atypical chemokine receptor ACKR3 leading to CXCL11 internalization which could represent a way of regulating CXCR3-mediated responses [93]. However, the precise role of CXCL11 in atherosclerosis has not been addressed so far. CXCL9 and CXCL10 both promote inflammation by inducing T cell polarization into Th-1/Th-17 cells, whereas CXCL11 drives the development of Treg cells to counter regulate inflammation [88]. Interestingly, the loss of CXCR3 via targeted deletion or pharmacological inhibition resulted in reduced plaque formation, diminished recruitment of Th-1 cells and increased migration of Tregs to lesions of ApoE^−/−^ mice receiving a WD for 10 weeks [94,95]. Furthermore, ApoE^−/−^ CXCL10^−/−^ mice fed a WD for either 6–12 weeks exhibited reduced atherogenesis and greater numbers of Treg cells in the lesion [96], while antibody-mediated CXCL10 inhibition in ApoE^−/−^ mice increased plaque stability [7]. In line with this, CXCL10 is associated with the development of vulnerable plaque in humans [97]. Taken together, interfering with CXCR3 and CXCL10 seems to exert atheroprotective function while the full picture of how this receptor mediates effects induced by its different ligands still needs further investigation.

### 1.10. CXCL12-CXCR4/ACKR3

CXCL12 (SDF-1) is described as a ‘primordial’ chemokine due to its essential roles in homeostasis and development, such as leukocyte homing and recruitment, stem cell recruitment as well as vascularization [98,99,100,101]. This chemokine signals through the receptors CXCR4 and ACKR3 (CXCR7), both of which are also essential for healthy cardiac development [102,103,104,105]. In 2007, GWAS analyzed chromosomal loci associated with CAD in humans and exposed novel loci which markedly contribute to CAD as well as myocardial infarction [106]. This study identified key genes that are of high importance in CVD and among those CXCL12 was one of the novel candidates. Other studies have also shown that circulating CXCL12 levels were associated with CAD severity as well as heart failure and its increase in circulation is recognized as a CVD risk factor [107,108,109].

Early studies examining the role of CXCL12 in vascular biology established that this chemokine promoted healing of the injured vascular tissue. CXCL12 was shown to boost in vivo EC progenitor recruitment in ischemic tissues via injections of CXCL12 locally to ischemic hindlimbs of mice. The findings led to the conclusion that CXCL12 endorsed ischemic neovascularization [110]. Further evidence from other studies showed that CXCL12 supported neointimal formation in carotid arteries as a result of CXCL12-dependent SMC recruitment in a wire induced vascular injury model using ApoE^−/−^ mice [111]. A recent study by Döring and colleagues dissected the role of CXCL12 in atherosclerosis by establishing a comprehensive cell-specific deficiency of the chemokine in ApoE^−/−^ mice [109]. The authors examined lesion areas in aortas as well as aortic arches in addition to macrophage and collagen content of the lesions in five different models of CXCL12 deficiency: ubiquitous knockout, hematopoietic knockout, non-hematopoietic knockout, SMC knockout, as well as EC-specific knockout. Whilst systemic deficiency of the chemokine did not affect lesion size or composition, its non-hematopoietic deficiency improved atherosclerosis. This effect was then mirrored by the EC-specific knockout of the chemokine, which decreased lesion sizes in the aortas as well as the aortic arches whereas increasing the collagen content within the lesions. This outcome was observed along with a systemic decrease of circulating CXCL12 levels leading to the conclusion that EC-derived CXCL12 promotes atherosclerosis [109].

The importance of the CXCL12–CXCR4 axis in vascular remodeling was shown by identifying its contribution to neointimal hyperplasia through recruitment of bone marrow derived SMC progenitors [112]. The CXCL12-CXCR4 axis was also studied in the context of atherosclerosis with a focus on CXCR4 using diet-induced atherosclerosis mouse models [20]. According to the study, Cxcr4 deficiency in the bone marrow of ApoE^−/−^ mice increased atherosclerotic lesion sizes and constant blockade of systemic CXCR4 via its antagonist AMD3465 led to higher numbers of circulating leukocytes, especially neutrophils, leading to a higher content of neutrophils within atherosclerotic lesions. Furthermore, AMD3465 treated mice had significantly less SMCs in their lesions. Thus, the authors concluded that interfering with the CXCL12–CXCR4 axis enhanced neutrophil recruitment to atherosclerotic lesions, which then stimulated lesion growth and instability. These results pointed toward a protective role of CXCL12-CXCR4 in atherosclerosis. It is worth mentioning, however, that this study did not specifically target the ligand induced effect of CXCR4, rather the deficiency of CXCR4 itself. Apart from CXCL12, macrophage migration inhibitory factor (MIF) can also bind to CXCR4 and the findings of this study can therefore not be attributed to a specific ligand of CXCR4. A study targeting this gap was published by Akhtar et al. in 2013 [113]. The authors partially ligated carotid arteries of *ApoE*^−/−^ mice and repeatedly injected CXCL12. They observed that the CXCL12 injections led to more stable plaque phenotypes compared to control mice; lesional macrophage content was decreased, whereas SMCs and collagen content was increased along with thicker fibrous caps. Interestingly, CXCL12 injections did not have an impact on lesion sizes per se. However, it should be mentioned that lesions of ligated arteries are phenotypically different from those of normal vessels.

The role of CXCR4, specifically in the artery, in a diet induced atherosclerosis mouse model was further assessed by Döring et al. using cell specific deletions of the receptor in endothelial cells and SMCs [114]. In contrast to their findings regarding EC-specific CXCL12, EC-specific CXCR4 was shown to limit atherosclerosis by supporting endothelial barrier function. According to their study, EC-specific deficiency of the receptor led to arterial leakage and thereby promoted arterial leukocyte invasion. Furthermore, their study established an association of a C-allele at rs2322864 within the *CXCR4* locus with increased risk for CHD in a regression analysis using human data from 92,516 CHD cases and 167,280 controls [114]. Another study investigating the effects of the CXCL12–CXCR4 axis in a hypercholesterolemia mouse model using black 6 (BL6) mice and revealed that mice fed with high fat diet had higher levels of CXCL12 in their circulation along with leukocytosis [115]. This finding was similar to the results of human studies, which the authors thought to be a result of disturbed CXCL12-CXCR4 interaction in the bone marrow due to high cholesterol levels. They have also shown that LDL treatment of human umbilical vascular endothelial cells (HUVEC) led to increased CXCL12 production [115]. Others have also revealed evidence linking the CXCL12 receptor ACKR3 to lipid metabolism [116]. Through genetic ablation of ACKR3 in mice with a wire induced vascular injury as well as ACKR3 ligand (CCX771) treatment in ApoE^−/−^ mice on high fat diet, Li et al. showed that ACKR3 promoted cholesterol uptake into adipose tissue and thereby controlled systemic lipid levels. However, this study does not disclose through which biological ligand ACKR3 exerted these effects.

Monocyte to macrophage differentiation is a key step supporting macrophage driven oxLDL clearance in the arterial intima, which eventually leads to the aggregation of foam cells and therefore fatty streaks. Interfering with this step by means of preventing macrophage accumulation may be a key to inhibit fatty streak formation. Therefore, key regulators of monocyte to macrophage differentiation can be viewed as a therapeutic target to impede atherogenesis. In an in vitro study, CXCL12 was shown to be secreted by human monocytes and the inhibition of either CXCR4 or ACKR3 via their antagonists significantly decreased CD163 expression of monocytes [5]. Moreover, CXCL12 treatments showed a decrease in RUNX3 and TGF-ß transcription in these cells, whereas CXCR4 antagonism through AMD3100 allowed RUNX3 expression. These findings indicate that monocyte-derived CXCL12 controls monocyte to macrophage differentiation in an autocrine manner by down-regulating the transcription factor RUNX3. These findings need further confirmation in in vivo models. Mice lacking CXCL12 specifically in the monocyte subset may be a good tool to study the impact of CXCL12 on cell differentiation. 

Evidence on CXCL12 suggests a pro-atherosclerotic role of the chemokine, whereas its receptors seem to play varying roles. Further studies are needed to improve the understanding of each axis in atherosclerosis. Whilst CXCL12 can bind two different receptors, its receptors can also be activated by additional ligands which increases the complexity and complicates conclusions to be drawn. Moreover, cell-specific effects of these axes vary, which is yet another strain in the path to map the roles of these chemokine-receptor interactions in this multidimensional disease.

### 1.11. CXCL16-CXCR6

CXCL16 is an unusual chemokine as it contains a mucin-like stalk, and transmembrane and cytoplasmic domains not found in other CXC chemokines [117]. The mucin-like stalk is also susceptible to cleavage releasing the chemokine domain, which acts to chemoattract CXCR6-expressing T, NK, NKT, B, and dendritic cells [117]. CXCL16 is expressed on stimulated ECs and SMCs, macrophages, DCs, and platelets, whereas its receptor CXCR6 is expressed on leukocyte subtypes [118,119]. It has been suggested that CXCR6 is a reliable marker of IFNɣ producing effector CD4 T cells, which drive Th1-mediated diseases like atherosclerosis [120]. In an ApoE^−/−^ mouse model where CXCR6 was replaced with a green fluorescent protein CXCR6^GFP/GFP^, a decrease in atherosclerosis was observed when compared with ApoE^−/−^ mice [121]. This reduction in atherosclerosis was attributed to a reduction of CXCR6^+^ T cells within the aortas. Further experiments demonstrated that CXCR6 is necessary for the recruitment of CXCR6^+^ leukocytes into the lesions. This reduction of CXCR6^+^ T cells within the aortas lead to a weakened production of INF-γ and a reduction of CD11b^+^/CD68^+^ macrophages in the aorta. CXCL16 is involved in multiple phases of the immune response, from antigen recognition to migration and infiltration of immune cells into sites of inflammation, including the atherosclerotic plaque. CXCL16 is also expressed on the plasma membrane directly mediating adhesion to cells expressing the receptor CXCR6 [118]. This cell-to-cell adhesion could conceivably facilitate the development of plaques in atherosclerosis. In a recent report it has been shown that platelets from the blood are able to adhere to the inflammatory chemokine CXCL16 expressed by the endothelium [122]. Hence, CXCL16 as well as platelet CXCR6 act as potent peripheral blood mononuclear cell adhesion ligands to the atherosclerosis prone vessel wall and thus promote the progression of atherosclerosis [123].

CXCL16 may also directly play a role in the development of foam cells as the expression of CXCL16 has been found to be upregulated in lipid-laden intimal macrophages and SMCs [124]. Originally, CXCL16 was identified due to its ability to scavenge phosphatidylserine and oxLDL and thus was originally considered to be a scavenger receptor [124,125]. Recently, CXCL16 has been shown to act as a scavenger receptor for oxLDL uptake on hepatic cells and results in the formation of foam cells in Non-alcoholic fatty liver disease [126]. Macrophages are not the only cell type to develop into foam cells, SMCs are also capable of accumulating lipids and take on a foamy appearance. In human atherosclerotic lesions CXCL16 is expressed in SMCs and it has been demonstrated that IFN-ɣ is a potent CXCL16 inducer of itself in human aortic SMCs in vitro [127]. IFN-ɣ induction of CXCL16 correlated with an increased uptake of oxLDL into these cells [127]. Therefore, it can be concluded that in vivo CXCL16 could facilitate the development of SMC foam cells, but this warrants further investigation. 

CXCL16 is also expressed on HUVECs and LPS induces the overexpression of CXCL16 along with TLR4, NF-κB and miR-146a but this induction can be blocked by the TLR4 inhibitor TAK-242 [128]. Additionally, either the overexpression or inhibition of miR-146a either inhibited or increased the LPS-induced expression of CXCL16, TLR4 and NF-κB protein production, respectively. Finally, the miR-146a-induced expression of CXCL16 was blocked by TAK-242. Thus, in HUVECs LPS stimulates CXCL16 expression via the TLR4/NF-κB signaling pathway, and simultaneously, miR-146 negatively regulates LPS-induced CXCL16 expression through a TLR4-dependent mechanism. An in vivo study demonstrated that the expression of CXCL16 was upregulated in atherosclerotic ApoE^−/−^ mice fed a high fat diet, compared with control ApoE^−/−^ mice fed a normal diet [129]. The expression levels of TRL4, IL-1 receptor-associated kinase 1, TNF-α, NF-κB, and IL-1β were also significantly upregulated in atherosclerotic ApoE^−/−^ mice compared with control mice. However, the expression of miR-146a and miR-146b was significantly downregulated in atherosclerotic ApoE^−/−^ mice compared with control ApoE^−/−^ animals. This suggests that CXCL16 may regulate the TRL4/NF-κB/CXCL16 signaling pathway, and that miR-146a and miR-146b may negatively regulate CXCL16 via TRL4/NF-κB signaling. However, further investigations in vivo are needed to identify the therapeutic potential of treating atherosclerosis via the inhibition of either TLR4 or miR-146a.

Previous clinical studies demonstrated that the serum levels of CXCL16 increase during atherosclerotic ischemic stroke and are higher in patients who exhibit microembolic signals compared to microembolic signals-negative patients [130,131]. In another clinical study 43 patients with end stage renal disease (ESRD) were separated into either the control group or the inflamed group based on their plasma C-reactive protein (CRP) levels; a control (CRP < 3.0 mg/L) and an inflamed group (CRP ≥ 3.0 mg/L) [130,132]. The inflamed group presented an increase in both MCP-1 and TNF-α protein expression and macrophage infiltration in radial arteries. Additionally, there was a significant increase in foam cell formation in the radial arteries of the inflamed group compared to the control. Immunohistochemical and immunofluorescence staining of these radial arteries of the inflamed group further showed that protein expression of CXCL16, CXCR6, and ADAM10 was increased. CXCL16 expression also correlated with P2X7R expression in the radial arteries of ESRD patients. This suggests that inflammation contributes to foam cell development in the radial arteries of ESRD patients via activation of the CXCL16/CXCR6 pathway, and is possibly regulated by P2X7R. Therefore, P2X7R may as well be a potential therapeutic target for atherosclerosis.

### 1.12. CX3CL1-CX3CR1

CX3CL1 and its receptor CX3CR1 are important for immune cell trafficking [133]. CX3CL1 is expressed as a membrane bound protein (mCX3CL1) the extracellular domain can be cleaved and released to act at distal sites [134]. CX3CL1 acts as a monocyte survival molecule as CX3CL1 and CX3CR1 deficient mice had a significant reduction of Gr1^low^ blood monocyte levels under both steady-state and inflammatory conditions [135]. Use of the Bcl2 transgene restored the wild-type phenotype, providing genetic evidence that the CX3CR1/L1 axis provides an essential cell survival signal [135]. Thus, loss of either CX3CL1 or CX3R1 could lead to monocyte apoptosis thereby effectively reducing the development and even regression of atherosclerotic lesion formation. CX3CL1 upregulation has also been observed in macrophage-rich human coronary artery plaques and several studies have demonstrated that genetic deletion of either CX3CL1 or CX3CR1 protects mice against atherosclerosis [135,136,137]. This suggests CX3CL1 plays a pivotal role in atherosclerosis both in the early and late stages of the disease. During atherosclerosis, monocytes expressing CX3CR1 bind and adhere to the endothelium, which expresses mCX3CL1 [138]. Certainly, CD16^+^CX3CR1^HIGH^ monocytes enhanced endothelial STAT1 and NFκB p65 phosphorylation resulting in an upregulated expression of CX3CL1 and IL-1β, and ICAM1 and VCAM1, compared to classical CD14^+^ monocytes [139]. This describes the mechanism by which CX3CR1 and its ligands can increase cardiovascular risk. Furthermore, a recent study has highlighted a novel model of CD8 T cell involvement in atherosclerosis. Whereby CX3CL1 and IL-15 synergistically activate the vascular endothelium to promote infiltration of CX3CR1^+^ memory CD8 T cells which further promote endothelial inflammation [140]. This interaction may be prevalent in aging and in HIV populations where circulating activated CX3CR1^+^ CD8 T cell numbers are often higher.

Treatment of Ldlr^−/−^ mice with a CX3CL1-Fc fusion protein, which interferes with the CX3CR1-CX3CL1 interaction, significantly reduced atherosclerotic lesions size independent of changes in cholesterol levels [141]. The same study demonstrated that in vitro using HUVEC, CX3CL1-Fc interferes with monocyte binding and in vivo it reduces leukocyte rolling and adhesion in murine capillaries [141]. This leads to a decrease in M1-like macrophages and T cells in the aortic wall, potentially decreasing chronic inflammation in the lesions. Hence, the long-acting CX3CR1 agonist prevents monocytes from adhering to the endothelial wall thereby reducing lesion formation offering potential therapeutic options.

Another study examined the potential therapeutic value of targeting vascular CX3CL1/CX3CR1 [142]. They transplanted the aortic segments from Cx3cr^−/−^ApoE^−/−^ into ApoE^−/−^ mice which resulted in reduced atherosclerotic plaque formation compared to ApoE^−/−^ or Cx3cr1^−/−^ApoE^−/−^ mice after receiving transplantation of ApoE^−/−^ aortas. This suggests that CX3CR1 on vascular cells plays a key role in plaque formation and progression. Recently a DNA vaccine, which targets CX3CR1 in ApoE^−/−^ was tested and resulted in an induction of anti-CX3CR1 antibodies [143]. Vaccinated mice exhibited a significantly reduced atherosclerotic plaque in the brachiocephalic artery. This was due to a reduction of macrophage accumulation and a reduction in lipid deposition, however there was no change to the M1 phenotype predominantly found in inflamed lesion. This suggests that the vaccine mainly limited macrophage infiltration. Overall, targeted DNA vaccination to CX3CR1 was well tolerated by the mice, inducing a strong immune response and attenuation of atheromatous plaque size. Overall, vaccination against certain chemokines may offer some therapeutic potential for the treatment of atherosclerosis.

### 1.13. MIF-CXCR2/CXCR4/ACKR3

Macrophage migration inhibitory factor (MIF) is now well known to be a pro-atherogenic cytokine which is also characterized as an atypical chemokine and it binds multiple chemokine receptors: CXCR2, CXCR4, and ACKR3 [144]. A study in rabbits demonstrated that MIF is upregulated in atherogenesis and later it was established that MIF is present in human atherosclerotic lesions with regards to various cell types [144,145].

An in vivo study revealed that monoclonal antibody based blocking of MIF in ApoE^−/−^ mice reduced aortic intimal macrophage content in addition to circulating inflammatory molecules, such as IL-6 [146]. Furthermore, local expression of inflammatory molecules in the aortic wall, such as ICAM-1, IL-12, and TNF-α, were also reduced in MIF antibody treated mice. Although these findings are known to significantly influence atherosclerotic lesion development, MIF blockade did not significantly alter lesion sizes compared to control mice in this study, despite a decrease. Another study, however, studying genetic ablation of MIF in Ldlr^−/−^ mice disclosed a highly significant reduction in atherosclerotic lesion sizes in the abdominal aortas of MIF deficient mice along with decreased intimal thickening in aortic arches [147]. Consistent with these findings, another study reported reduced vascular inflammation and neointimal thickening upon neutralizing MIF in an atherosclerotic mouse model with experimental angioplasty [148]. In addition to vascular inflammation, MIF was also demonstrated to influence plaque stability after vascular injury in *ApoE^−/−^* mice. Schober et al. reported reduced foam cell content along with an increase in smooth muscle cell content as well as collagen in the lesions of mice treated with neutralizing monoclonal MIF antibody [149]. Via in vitro flow assays, the authors further confirmed that MIF treated aortic endothelium exhibited increased monocyte recruitment and oxLDL driven monocyte arrest could be inhibited by monoclonal antibody treatment, suggesting that endothelial MIF mediates oxLDL triggered monocyte arrest. In addition to CXCL1, MIF can also signal through CXCR2 and it has also been shown to mediate monocyte arrest through CXCR2 [150]. Additionally, this study has also reported that immobilized MIF on aortic endothelial cells can elicit effector T cell arrest, which could be inhibited by CXCR4 blockade. Moreover, MIF is also shown to mediate monocyte chemotaxis through CXCR2 as well as B-cell migration through ACKR3 [150,151]. Collectively, these data demonstrate the spectrum of numerous inflammatory roles of MIF which significantly contribute to atherosclerosis and therefore the importance of this chemokine as a therapeutic target.

## 2. Recent Highlights and the Road Ahead

Chemokines and their receptors are being studied continuously in the research of atherosclerosis. Although numerous axes still remain to be elucidated, the quantity of studies dissecting the roles of various chemokine-receptor axes is fairly rich. Therefore, it is necessary to recapitulate these findings in a condensed as well as an up-to-date manner. In this review, we highlighted several new studies which provided significant evidence advancing the research of chemokine function in atherosclerosis. For example, CCL2 was recently shown to be released onto the arterial endothelium rhythmically by myeloid cells oscillating in a circadian fashion [16]. This finding presented a novel approach called ‘chrono-pharmacological treatment strategy’ targeting the peaks of pro-atherosclerotic myeloid cell action. Taken further this study emphasizes to reschedule complicated surgeries to times where less CCL2 is released. Another recent mouse study presented protective and anti-inflammatory effects of MKEY (a specific peptide blocking the CCL5-CXCR4 interaction) in a myocardial ischemia/reperfusion injury model and suggested that it could be used therapeutically in atherosclerosis and myocardial infarction treatment [36]. A human study investigated the impact of CCR5 antagonist maraviroc in patients with HIV on several cardiovascular risk markers and found these to be reduced, suggesting a need for further studies on maraviroc’s effects on CVD [40]. Further, An et al. showed that CXCL8 contributes to atherosclerosis development by driving neutrophil extracellular trap (NET) formation via CXCR2 [85]. The study also showed that CXCR2 inhibition successfully abrogated NET formation in human neutrophils, proposing that neutrophil specific interference with CXCR2 could hold therapeutic options. The association between CXCL12 and CAD [107] was also carried further by the findings that endothelial deficiency of CXCL12 decreased atherosclerotic lesion sizes in mice whilst increasing lesion stability through lesional collagen content, establishing a pro-atherosclerotic role of endothelial CXCL12 [109]. Further, examining the impact of CX3CR1–CX3CL1 axis inhibition via a soluble CX3CR1 agonist revealed improved atherosclerosis via the inhibition of monocyte-endothelial cell interaction and suggested this approach as a treatment strategy for atherosclerosis [141]. Taken together following up on chemokine/chemokine receptor axis in chronic vascular inflammation still reveals promising and future-orientated insights and mechanisms which may allow for therapeutic targeting to utilize treatments to dampen chronic inflammation in CVD as an addition to lipid lowering therapies.

## 3. Conclusions

Chemokines are essential in orchestrating the development of atherosclerosis, from the initial stage of endothelial dysfunction, chemokines like CXCL1 act as a chemoattractant for several immune cells (Figure 1). After aiding the homing of these cells to the lesions, chemokines like CXCL8 activate the endothelial barrier by increasing adhesion molecules to allow the infiltration of various immune cells. Moreover, chemokines like CXCL16 directly act as adhesion molecules to the CXCR6 expressed on platelets. Even after immune cells have infiltrated into the intima chemokines then dictate their phenotype, like CXCL4 which establishes the unique M4 macrophage phenotype, promoting the development of atherosclerosis (Figure 2). Foam cell development, a key feature of atherosclerosis, is also controlled by chemokines as for example CXCL16 directly facilitates the development of foam cells from macrophages acting as scavenger ligands for oxLDL (for an overview of the murine studies please also refer to Table 1).

Nevertheless, future work is necessary to understand the role of CXCL16 in the formation of foam cells from SMCs. Finally, establishment of a fibrous cap around the lesions to ensure stability and prevent rupture may be influenced by CXCL10 concentrations which impact on fibrous cap thickness. Taken together, many aspects of chemokines and their role within atherosclerosis have still to be answered, such as the receptor for CXCL4 still remains elusive. 

With respect to therapeutic targeting one has to keep in mind that receptors can be activated by many chemokines in some cases with varying outcomes. Cell specific therapeutic targeting of specific chemokines may be therefore the most promising approach in tackling atherosclerosis. Thus, chemokines continue to be an exciting avenue of research with potential real-world implications in atherosclerosis treatment.

## Figures and Tables

**Table 1:** Overview of the inflammatory chemokines and their role in murine atherosclerosis

Chemokine axis	(Mouse) Model	Treatment	Outcome	Ref.
**CCL2-CCR2**	C57/Bl6	Thioglycollate (i.p.) injection	Reduced monocyte recruitment	[11]
	CCL2^−/−^ Ldlr^−/−^	12 weeks WD	Smaller lesions, reduced recruitment	[12]
	CCL2^−/−^ Apoe^−/−^ & CCL2^−/−^ bone marrow into Apoe3 Leiden mice	5–13 weeks WD	Smaller lesions, reduced recruitment	[13,14,15]
	Apoe^−/−^	4 weeks WD, CCR2 Antagonist RS102895	Smaller lesions, less macrophages	[16]
	CCR2^−/−^ Apoe^−/−^	WD, Cuff placement	Reduced neointimal formation	[17,18,19]
**CCL3**	Ldlr-/ transplanted with CCL3^−/−^ BM	12 weeks WD	Reduced lesion size	[25]
	Apoe^−/−^	Atorvastatin, 16 weeks WD	Atorvastatin blocks CCL3 expression and thereby lesion formation	[26]
**CCL5-CCR1/CCR3/** **CCR5**	Apoe^−/−^	Wire injury, inhibition of CCL5	Reduced neointima formation	[29]
	Apoe^−/−^	14 weeks WD, Met-Rantes (CCL5 inhibitor)	Smaller lesions and reduced lesional number of macrophages and T cells	[30]
	Apoe^−/−^	CCL5-CXCL4 heterodimer blocking with MKEY	Reduced scar formation in MI	[36]
	CCR5^−/−^ Apoe^−/−^	WD	Reduced lesion size	[21]
	CCR1^−/−^ Apoe^−/−^	WD	Increased lesion size	[21]
	CCR1^−/−^ Apoe^−/−^	4 weeks WD	Reduced lesion size	[38]
**CCL17**	CCL17^e/e^ Apoe^−/−^	4 and 12 weeks WD	Reduced lesion size, increased number of Treg	[44]
	Apoe^−/−^	WD, CCL5-CCL17 heterodimer inhibition	Smaller lesions	[47]
**CCL19, CCL21/CCR7**	ApoE^−/−^CCR7^−/−^	8 weeks WD	Increased lesion size and T cell number	[60]
	Ldlr^−/−^CCR7^−/−^	12 weeks WD, adoptive transfer of wild type T cells	Increased lesion size	[61]
	CCL19^−/−^ CCL21^−/−^BM into Ldlr^−/−^	WD	Increased plaque stability, no change in lesion size	[62]
**CXCL1-CXCR2**	Cxcl1^−/−^ Ldlr^−/−^	16 weeks WD	Reduced lesion size, less macrophages	[65]
	Cxcl1^−/−^ BM into Ldlr^−/−^	16 weeks WD	No effect	[65]
	Cxcr2^−/−^ BM into Ldlr^−/−^	16 weeks WD	Reduced lesion size, less macrophages	[66]
	Apoe^−/−^	8 weeks WD versus chow diet	Increased levels of CXCL1 in the serum, more monocytes in the circulation	[38]
	Apoe^−/−^	CXCL1 neutralization with antibody injections, 4 weeks WD	Reduced lesion size, decreased monocyte mobilization	[38]
**CXCL4**	PF4^−/−^ Apoe^−/−^	WD	Reduced lesion size	[33]
**CXCL8**	Apoe^−/−^	Injection of human CXCL8 analog, WD 12 weeks	Reduced lipid levels, reduced lesions?	[81]
**CXCL12-CXCR4/ACKR3**	Nude mice	Injectionof CXCL12 into hindlimb of mice	Induces ischemic neovascularization	[110]
	Apoe^−/−^	Vascular injury	SMC-derived CXCL12 mediates neointima formation	[111]
	Apoe^−/−^ lacking CXCL12 specifically in ECs	WD 12 weeks	Reduced lesion formation	[109]
	Ldlr^−/−^ with CXCR4^−/−^ BM, or Apoe^−/−^ with CXCR4 blocking	WD, AMD3465	Enhanced lesion formation	[20]
	Apoe^−/−^	WD and repetitive CXCL12-injections	More stable plaque phenotype in partial ligation, no size differences	[113]
	Apoe^−/−^ lacking CXCR4 in ECs or SMCs specifically	WD 12 weeks	Enhanced lesion formation, higher permeability	[114]
	C57/Bl6	4 weeks high cholesterol diet	Higher CXCL12 levels	[115]
	ACKR3^−/−^ Apoe^−/−^	Wire induced vascular injury, WD	Enhanced peripheral cholesterol levels, increased neointima	[116]
**CXCL16-CXCR6**	CXCR6^GFP/GFP^ ApoE^−/−^	WD	Reduced lesion size, less T cells and macrophages	[121]
	Apoe^−/−^	WD versus Chow diet	Upregulation of CXCL16 under WD	[125]
**CX3CL1-CX3CR1**	Apoe^−/−^ with CX3CR1^−/−^ BM	12 weeks WD	Induces monocyte apoptosis, reduces lesion size	[135]
	CX3CL1^−/−^ Apoe^−/−^ CX3CL1^−/−^ Ldlr^−/−^	~12 weeks WD	Reduced lesion size and monocyte recruitment	[136]
	Ldlr^−/−^	Injection of a long lasting FcCX3CL1 version, agonist, WD	Reduced lesion size and monocyte recruitment	[141]
	Apoe^−/−^	Transplantation of aortic segments from *Cx3cr^−/−^ApoE^−/−^* into *ApoE^−/−^*	Plaque regression	[142]
	Apoe^−/−^	DNA vaccine which induces antibodies against CX3CR1, chow diet	Reduced lesion size, less macrophage accumulation	[143]
**MIF-CXCR2/CXCR4/ACKR3**	Apoe^−/−^	MIF antibody blocking	Reduced macrophage load in lesions	[147]
	MIF^−/−^ Ldlr^−/−^	WD	Reduced lesion size	[148]
	Apoe^−/−^ (vascular injury)	MIF antibody blocking	Reduced inflammation and intimal thickening	[149,150]
